# Alcohol and Aquatic Activity: Young Males' Perceptions of Risk and Social Identity Through the Lived Experience

**DOI:** 10.1002/hpja.70045

**Published:** 2025-05-14

**Authors:** Stephanie R. Smith, Sabryna V. Sas, Jacob J. Keech, Amy E. Peden, Martin S. Hagger, Kyra Hamilton

**Affiliations:** ^1^ School of Psychological Sciences University of Tasmania, Inveresk Campus Launceston Tasmania Australia; ^2^ School of Applied Psychology Griffith University, Mt Gravatt Campus Brisbane Queensland Australia; ^3^ School of Population Health, Faculty of Medicine and Health UNSW Sydney Kensington New South Wales Australia; ^4^ Discipline of Public Health and Tropical Medicine James Cook University Townsville Queensland Australia; ^5^ Department of Psychological Sciences University of California Merced California USA; ^6^ Faculty of Sport and Health Sciences University of Jyväskylä. Liikunta Jyväskylä Finland; ^7^ Health Sciences Research Institute University of California Merced California USA

**Keywords:** alcohol, drowning, injury, lived experience, risk perception, social identity, swimming

## Abstract

**Introduction:**

This research explored the lived experiences, risk perceptions and social identity beliefs related to alcohol consumption around water among young Australian males.

**Methods:**

Purposive sampling was used to recruit 23 Australian males aged 18–30 who had previously consumed alcohol around water. An online survey collected demographics, eligibility and swimming ability, followed by qualitative telephone interviews. An interview guide developed based on extant literature and team member expertise gathered data on lived experience of alcohol consumption around water, risk perception and social identity. Interviews were recorded, transcribed verbatim and analysed using a reflexive hybrid thematic approach in NVivo.

**Results:**

With respect to lived experience, participants described consuming alcohol around water primarily as a social and unplanned activity, often occurring with friends and family and in locations lacking formal safety measures. In terms of risk perception, despite recognising the dangers of consuming alcohol around water, many underestimated personal risks and overestimated their risk assessment abilities. Concerning social identity, participants identified typical individuals engaging in these activities as young, predominantly male and sensation‐seeking, often viewed as socially undesirable and reckless. However, many did not see themselves as fitting this description, instead describing their behaviour as more cautious and responsible.

**Conclusions:**

This study provides valuable insights into how lived experiences, risk perceptions and social identities influence young Australian males' decisions to consume alcohol around water. *So what?*: The findings underscore the need for targeted public safety campaigns and interventions that leverage lived experiences and psychological insights to effectively reduce alcohol‐related risks in aquatic environments.

## 1 Introduction

Drowning represents a significant, yet preventable, public health issue that accounts for 7% of all deaths caused by injuries, making it the third leading cause of injury‐related mortality globally [1]. Males are disproportionately represented in drowning incidents [2, 3, 4], with alcohol and drug use identified as key risk factors in drowning among young men [5, 6, 7]. Despite its importance, few studies have focused on preventive measures aimed at reducing alcohol use by young people in aquatic environments [8]. Given drownings from alcohol are often preventable and commonly occur among males, it is important to identify the beliefs young males hold for this important water safety behaviour which can then serve as a useful source of strategies to target in future interventions.

There is a limited, yet growing, body of research examining the subjective experiences, knowledge and attitudes of males towards alcohol consumption around water [9, 10, 11, 12]. For example, recent qualitative and mixed‐methods research has applied the theory of planned behaviour [13] to identify key behavioural (expected outcomes), normative (significant others' approval/disapproval) and control (facilitators and barriers) beliefs that underlie alcohol consumption around water in samples of young adults from Australia and the United Kingdom [12], and specifically among young Australian males with a history of drinking and swimming [14]. This research offers valuable insights into the beliefs held by young males towards alcohol use around water, offering a basis to inform the development of focused prevention strategies that address these key beliefs. However, expanding beyond a single theoretical framework is essential to incorporate a wider range of psychological constructs, thus enhancing our understanding of the complex beliefs and cognitions that drive decision‐making and behaviour.

The current study builds on existing theoretical exploration within this context [12, 14], examining additional factors such as lived experiences, which can provide insight into how individuals' personal histories and situational contexts may shape their drinking behaviours around water; risk perception, which explores young males' assessment of the potential dangers associated with drinking in these settings; and social identity, which examines the role of group norms and social affiliations in shaping individual choices, particularly within peer‐driven environments. These constructs may be particularly pertinent among young males, a group that frequently consumes alcohol around water and whose behaviour is influenced by risk perceptions and the presence of peers [8]. Young males may experience unique pressures to conform to group norms and may underestimate or normalise the risks associated with alcohol consumption in aquatic environments, making these constructs essential for understanding the motivations behind their behaviours. Together, these factors offer a more nuanced understanding of alcohol consumption motivations in this high‐risk group, directly informing targeted interventions that address their specific experiences, risk perceptions and social identity.

*Lived experience* involves understanding the context of behaviours, such as consuming alcohol around water, through the lens of those who have personally engaged in these activities [15]. This approach is important for capturing the complex interplay of individual perspectives and situational factors that influence risky behaviours. Moreover, research has demonstrated that past experiences of drinking and swimming are predictive of future intentions among young Australian men to engage in such activities [10]. Gathering insights from individual experiences is a valuable step for crafting targeted prevention strategies that resonate with at‐risk groups [16], enhancing intervention effectiveness and ultimately preventing drowning‐related incidents. The current study, therefore, focused on understanding the consumption of alcohol around water through the perspectives and experiences of males who had previously engaged in this behaviour.

*Risk perception* refers to the subjective perceptions or beliefs regarding the potential dangers (i.e., perceived severity) and the likelihood of adverse outcomes (i.e., perceived susceptibility) associated with engagement in a behaviour, serving as a motivational force for the adoption of preventative behaviours and refraining from risk‐taking behaviours [17]. Risk perception is a key component of theoretical frameworks such as the health belief model [18] and the health action process approach [19]. Understanding these perceptions is particularly relevant in scenarios where behaviours—such as alcohol consumption around water—pose significant health threats, as research suggests that risk perception plays an essential role in behavioural decision‐making within higher risk contexts [20]. This importance is further underscored by a recent scoping review identifying risk perception as a key factor in young people's alcohol use around water [8]. By qualitatively examining how participants perceive and interpret these risks, this study contributes in‐depth insights into the nuanced ways risk perception shapes behavioural decisions in aquatic environments among males with prior experience in this behaviour.

*Social identity* relates to an individual's self‐concept, which develops as a result of belonging to a particular social group [21]. Exploring social identity in the context of males' alcohol consumption around water can shed light on the role of group norms in shaping behaviour. Theories like social identity and self‐categorisation suggest that individuals often adopt behaviours that align with the norms of their social groups, especially when these norms are prominent [22, 23]. Research supports the role of social dynamics in drinking behaviours, with a recent meta‐analysis finding that peer group identification is positively associated with alcohol use [24]. In male groups where alcohol consumption is viewed as acceptable or even encouraged, social alignment with these norms could increase conformity to potentially risky behaviours, such as drinking around water. Additional literature suggests that peer presence, social acceptability and group settings significantly increase the likelihood of alcohol consumption, even when individuals are aware of the risks [24]. Furthermore, in the specific context of aquatic settings, a recent study identified the presence of others—particularly peers—as a contributing factor in young people's decisions to consume alcohol around water [8]. To further investigate these social dynamics, this study draws on in‐depth qualitative data to examine how social identity influences drinking behaviour around water in young males with prior experience, offering insights to guide strategies that leverage group influence towards safer practices in aquatic environments.

This qualitative study aimed to explore the lived experience, risk perceptions and social identities of young males regarding alcohol consumption around water. By focusing on the firsthand experiences of this at‐risk group, the study provides a unique understanding of the motivations, perceptions and social influences underpinning alcohol consumption around water. The insights gained are expected to inform further empirical research and the development of targeted strategies to promote responsible drinking practices among young males around water, ultimately aiming to reduce drowning incidents and other alcohol‐related harms.

## 2 Method

### 2.1 Participants

A purposive sampling method was used to recruit Australian males aged 18–30 years who met the experience criteria of having previously consumed alcohol in, on, or around water. Social media posts, university broadcast emails and online media releases were used to recruit participants. An AU$50 department store gift card was provided to participants upon completion of the interview. All participants who agreed to participate remained in the study. Data were collected during September and October 2020.

### 2.2 Design and Procedure

The study was conducted and reported in accordance with the Consolidated Criteria for Reporting Qualitative Research (COREQ) guidelines [25]. This project received ethical approval from the University Human Research Ethics Committee (ref no: 2019/294). Participants received an information sheet detailing the study. After consenting, participants completed a brief online demographic survey which collected data on age, gender, postcode, marital status, highest education level, household income and current swimming ability (i.e., self‐reported ability level and achievable swimming distance without stopping). Following this, a semi‐structured telephone interview was conducted, comprising a range of open‐ended questions. Only the interviewer (Author 2) was present at their location during the interview, and participants were encouraged to choose a private and quiet place for the interview. The interviews lasted approximately 30 min. The interviewer has university qualifications in psychology and criminology and is trained in qualitative methods and interview techniques. At the time of the study, the interviewer was a research assistant and PhD candidate. There was no prior relationship between the interviewer and the participants, and the participants were informed of the interviewer's role and the study's purpose before starting the interview.

Participants were asked to openly share their experiences and beliefs surrounding the consumption of alcohol around water, with minimal interruptions and occasional prompting to allow them to speak freely when answering questions. The interviewer employed in‐interview member checking techniques, including confirming summaries, to ensure understanding and minimise bias. Interviews continued until all interested and available participants had been interviewed, and no repeat interviews were conducted. A reflexive journal was kept by the interviewer to reflect on assumptions and maintain transparency in analysis.

### 2.3 Data Analysis

Interviews were audio‐recorded and transcribed verbatim, removing any identifying data and assigning pseudonyms. Participants were not provided with interview transcripts, as interviewer understanding was checked throughout the interview using confirming summaries. Participants were informed that they could contact the research team if they had any questions or concerns regarding the interview content. The analysis followed a reflexive, hybrid thematic approach guided by both deductive and inductive elements [26]. To structure the initial coding framework, we employed three broad categories—lived experience, risk perception and social identity—derived from the interview protocol. These categories provided an overarching, deductive scaffold. Within each category, coding proceeded inductively, allowing data‐driven themes (e.g., the social nature of alcohol consumption around water, perceived ability to assess risk and evaluate own skills) to be derived. Thus, although the top‐level categories were informed by our research questions, the specific themes within them were generated from the data itself. This process was facilitated using NVivo software (Version 11.0). Author 2 coded all transcripts, and Author 1 independently coded 30% of the data to enhance analytic rigour. Throughout the process, we engaged in reflexive practice, acknowledging our roles and potential influences on the interpretation of findings. Themes were iteratively refined through team discussions to ensure credibility and coherence. Participants did not provide feedback on the findings.

### 2.4 Measures

The target behaviour was *alcohol consumption around water*. To investigate this behaviour, an interview protocol was created to elicit descriptions from young males about their decisions to consume alcohol around water. Interview questions were specifically designed to gather detailed accounts from young males about their lived experiences (i.e., personal histories and situational contexts related to drinking and swimming); perceptions of risk (i.e., assessments of the potential dangers associated with drinking in these settings); and social identity (i.e., the potential role of group norms and social affiliations in shaping individual choices, particularly in peer‐driven environments). The interview questions were developed by the authors in collaboration with Royal Life Saving Society–Australia. Questions were constructed for the current context based on prior research examining lived experience [15], risk perceptions, masculinity and male identity, including similarity and favourability from the prototype willingness model [27, 28], water safety [29, 30] and good principles in qualitative research [31].

#### 2.4.1 Lived Experience of Alcohol Consumption Around Water

Participants were prompted to discuss in detail the different occasions they have engaged in alcohol consumption around water. Participants were asked to describe the situation where this occurred, and any other factors they thought were important to describing the situation. Lived experience of alcohol use was assessed using one open‐ended question: ‘As you know, you are participating in this study because you mentioned that you have had alcohol around water in the past. Can you tell me about that, and about those experiences?’ This question also included probes about each time they consumed alcohol around water, their thoughts before, during, and after the event, and the context and any encouragement from others.

#### 2.4.2 Risk Perception

Risk perception was explored using one open‐ended question about the perceived level of risk associated with the behaviour, and any hesitancy towards engaging in the behaviour: ‘During situations where you have consumed alcohol around water, were there ever any thoughts about risk, or any hesitations?’ This question also included probes that built upon the previously discussed lived experiences of alcohol consumption around water. These probes explored participants' thoughts and feelings regarding risk, specific instances where they felt uncertain or worried, and whether their decision‐making or actions changed because of drinking alcohol around water.

#### 2.4.3 Social Identity

Social identify was explored using one open‐ended question about participants' perceptions of the types of people who consume alcohol around water: ‘When you think about the types of people that engage in alcohol consumption around water, what thoughts come to your mind?’ This question also included probes on whether these behaviours were characteristic of certain types of people or specific age groups, their social desirability, participants' identification with these groups, and their self‐perception when consuming alcohol near water.

## 3 Results

Twenty‐three young Australian males aged 18–30 years (*M* = 23, SD = 3.43) participated in this study. Most were from Queensland (91.3%), and the rest were from New South Wales (8.7%). The sample comprised current university students (56%) and community members (44%). A large proportion of participants reported coming from an English‐speaking background (78%), most were never married (95%), and nearly half had an undergraduate university degree (47%). Most participants rated their swimming ability as good to excellent (78%) and reported being able to swim 100 m or more without stopping (74%). Participant demographic characteristics are presented in Table 1. All males reported an awareness of the risks associated with swimming after consuming alcohol and described alcohol as a key contributor to the risk of drowning or injury around water. Various themes were identified in the males' descriptions about their decisions to consume alcohol around water. Themes were organised based on the guiding concepts of lived experience, social identity and risk perceptions. The most salient themes across participants are presented below. Extracts are indicated by participant number (e.g., P01). Table 2 provides a comprehensive selection of example quotes for each identified theme.

**TABLE 1:** Demographic characteristics of *N* = 23 males who have previously engaged in alcohol consumption around water.

Demographic characteristic	*N* (%)
Marital status	
Married registered	0 (0.0)
Married de facto	1 (4.3)
Separated/divorced	0 (0.0)
Widowed	0 (0.0)
Never married	22 (95.7)
Non‐English speaking background	
Yes	5 (21.7)
No	18 (78.3)
Employment status	
Full‐time work	3 (13.0)
Part‐time/casual work	8 (34.8)
Full‐time student	10 (43.5)
Part‐time student	3 (13.0)
Unemployed/home duties	5 (21.7)
Household income (annual)	
Nil–$18 200	8 (34.8)
$18 201–$37 000	3 (13.0)
$37 001–$80 000	8 (34.8)
$80 001–$180 000	4 (17.4)
> $180 001	0 (0.0)
Highest educational attainment	
Completed junior school (Year 10)	0 (0.0)
Completed senior school (Year 12)	7 (30.4)
TAFE certificate/diploma	3 (13.0)
Undergraduate degree	11 (47.8)
Postgraduate degree	2 (8.7)
Age	
Mean (SD)	23.43 (3.44)
Range	18–30
State of residence	
Queensland	21 (91.3)
New South Wales	2 (8.7)
Current swimming ability	
Very poor	0 (0.0)
Poor	0 (0.0)
Fair	0 (0.0)
Average	2 (8.7)
Good	9 (39.1)
Very good	6 (26.1)
Excellent	6 (26.1)
Achievable length of consistent swimming without stopping	
Cannot swim	0 (0.0)
Less than 25 m	0 (0.0)
25 up to 100 m	6 (26.1)
100 up to 200 m	4 (17.4)
200 up to 300 m	5 (21.7)
300 up to 400 m	0 (0.0)
More than 400 m	8 (34.8)

**TABLE 2:** Key themes relating to lived experience, risk perception and social identity among males who have previously engaged in alcohol consumption around water.

Theme	Quote
Lived experience of alcohol consumption around water	
Consuming alcohol around water usually happens with friends and family	– ‘So yeah probably earlier this year and around Australia Day me and some mates were all down by the pool, oh not the pool, by the beach sorry, having drinks as you do and playing a bit of sport as well like some beach cricket. And then yeah you get hot and then you go in to the water for a swim, there weren't any flags around where we were’. (P19) – ‘Friends or family yeah. Pool Party was friends, the boat was family, and then the beach was friends’. (P05)
We usually go swimming after partying	– ‘Well mostly things like pool parties. You know you invite a few of your friends around. Depends on kind of the size of the house and size of the pool and everything like that. And obviously your social circle yeah but you'd usually drink and then because in Australia it's so hot we just go to the pool. Yeah and you know you're not supposed to but a lot of us would bring like cans and things like that down the pool’. (P07) – ‘Usually it would just be if you're at a party and it happens to be close to the beach’. (P01)
Encouragement from friends	– ‘When we went camping, we all just kind of decided to do it. The first time I was with basically an equal group of guys and girls and the guys kind of egged each other on and I think only two of the girls went in and the rest of them were kind of like, “No don't”’. (P23)
Swim when there are no lifeguards or flags	– ‘Yeah but otherwise usually I end up swimming at beaches that don't have lifeguards like or at times when there aren't lifeguards’. (P08) – ‘I'd never swim at the beach if the flags weren't up. So because it was after a big night umm yeah’. (P13)
Swimming was not planned	– ‘Right well when I first saw the study I thought of two occasions specifically; one was at the beach when like, it was at Surfers Paradise, I guess it was kind of late, it would have been like nine or ten I think. And we were like were going to walk to a different club but then we ended up I don't know somehow going to the beach instead’. (P23) – ‘It might be a hot night of summer and someone decides to go for a walk to the beach and then people decide to go swimming you don't necessarily leave the party to go for a swim’. (P01)
Risk consideration	
Perceived ability to assess risk and consider own skills	– ‘I probably wouldn't have drunk alcohol and then went to the beach. But yeah in a commercial setting in a pool like that the risk of drowning was in my personal assessment very low’. (P05) – ‘Well I mean you know like from my standpoint it's like because I can swim and I know like my limits as well. So like I've always made sure that you know like I have the right amount of alcohol, not much where I just get super intoxicated, where I was like blind or something you know. Like I think that would be dangerous, you know like in the ocean. And making sure not to go too deep as well, just making sure that I was at the right, I suppose, at the shallow area’. (P16) – ‘it's happened to me before I definitely where I've thought “Oh I have had a few drinks I should probably just maybe not do, maybe not be jumping, I'll just sit by the edge or something.” Just a bit more relaxed I think rather than trying to swim fifty metres. Also don't feel like swimming fifty metres after I've had a few beers’. (P14) – ‘I wasn't too concerned just because the environment didn't look too bad. It [the ocean] was calm and everything and I wasn't too concerned with my abilities that day.’ (P19) – ‘I guess for us we were all wearing life jackets. We were all you know four drinks over three hours or four hours isn't really going to be too significant of an impact on how I think I'd be able to swim. Even prolonged swimming you know trying to stay afloat for you know ten, twenty minutes would be I think be fine’. (P05) – ‘Not personally. I have quite a high tolerance. I don't often get to the point where I'm so drunk that I can't swim or stand or anything like that. … I have a feeling if I was too drunk to swim I wouldn't get in the pool’. (P07)
Consideration of risk after the event	– ‘Usually not at the time… Yeah if I felt unsafe it was probably normally afterwards that it got thought of’. (P22) – ‘Definitely after the fact. At the time it's kind of just like a fun like “Oh let's just do this”… And then after you sort of like “Oh that was probably… probably shouldn't have done that it's probably a bit risky”’. (P06)
Risk not considered	– ‘Umm I don't think I've consciously thought “Oh I am a bit intoxicated I shouldn't swim, I should be more cautious.” But I think stuff like, I think you kind of know well I like to think I know where I'm at kind of thing’. (P14) – ‘I don't know that I specifically thought about it [risk]… when I'm sober I can look at it objectively and be like, “I shouldn't do that”… But just at the time I guess I kind of wrote it off as being a symptom of being young and stupid.’ – ‘Not really at the time it's kind of like in the moment… I guess it kind of feels like you've always heard that like drinking and swimming is kind of like drinking and driving and not exactly very safe but it just happens sometimes at that point in time it [risk] didn't come to the front of the mind’. (P17)
Social identity	
Characteristics of typical individuals who consume alcohol around water	– ‘Yeah especially in a social where there's an audience. So probably people who like attention, it's definitely probably something that a characteristic, a trait that someone would have who would be jumping off a roof at a party I'd say. Or being reckless around water whilst drinking’. (P14) – ‘That would probably just be someone that's adventurous. And yeah doesn't mind getting in cold water in the middle of the night or something like that. And they'll probably even realise the dangers but they just think that they're better than… That they'll… That there's nothing to worry about’. (P01) – ‘Like reckless, I suppose young, fun, what else like stupid… Just living life, I don't know’. (P16) – ‘I'd generally say sort of outgoing, extroverted type of people. Generally quite talkative or friendly or loud. Just those types of people I guess’. (P19) – ‘I guess maybe that might be show‐offs or they might take risks. They might take risks normally. So if they're around the pool they might yeah take extra risks or something’. (P03) – ‘Yeah more adventurous definitely. Especially when they're like not swimming exactly. Like boating or jet skiing or wakeboarding or anything like that? Yeah definitely more adventurous’. (P02)
Perceptions of social desirability	Negative perceptions: – ‘Probably a little bit stupid or you know probably just stupid’. (P11) – ‘I think they'd think they're like really foolish, unnecessary risk taking’. (P17) – ‘Not as very responsible people at all. Just irresponsible really. If I saw that behaviour around me I would certainly think it was very careless, risky that behaviour’. (P18) – ‘I think they probably a bit umm irresponsible. You might say. A danger to themselves yeah putting themselves at risk which is not great for other people who might really need the help’. (P10) – ‘Oh I think you know like generally I think people would not think too highly about it just like that, I think they'd see it as immature and reckless. And compulsive.’ (P13) – ‘I'd describe those behaviours as very careless. Very irresponsible for those people who are doing that and people that are heavily under the influence while around and maybe have a bad influence around them as well’. (P18) – ‘I think they think it's like obviously like a danger especially if you know families are around and they kind of want to protect their kids because they don't know what these people could do’. (P06) – ‘It's just irresponsible stupid you know careless. It's selfish as well I guess if you've consumed alcohol and you're taking control over something or have, you know power this or whatever. Then that's you know pretty selfish considering you do have people's lives in your hands’. (P04) Neutral or positive perceptions: – ‘But then other people might think: “Oh wow they're very adventurous and cool. Something like that”’. (P01) – ‘I think it depends how I guess relaxed, oh not relaxed how…how relaxed the individual is as in they probably don't see it as a big deal’. (P15) – ‘That's the funny thing because like it's socially acceptable for people to do you know like in some parts of the world. Like say for example in Mexico like that's like a normal occurrence so anyone like really mind, like them doing that’. (P16) – ‘Some might think they are very brave’. (P17) – ‘I'd probably say it's pretty Australian. I think it's like it's part of our culture, like it feels like an iconic Australian thing to do is have a barbie have some beers have a swim or like have a surf. And I don't know it's like… it's probably like the ideal environment for hanging out with your friends, I would say’. (P21) – ‘Because I think for a lot of people like for myself if what they were doing wasn't too dangerous I'd just sort of think that's normal behaviour. If you're young and by the pool why wouldn't you have a few drinks and goof around?’ (P03) – ‘Depending on what they're doing I think a lot of people would kind of disregard it’. (P05) Negative social desirability not directly related to drinking and swimming: – ‘I think if it's the type that are drinking heaps and getting really rowdy with a big bunch of people I think people would not perceive them really well. They'd probably annoy a lot of people, they annoy me a lot of the time if I'm there and they're getting too rowdy… But yeah I think those people are not looked upon favourably in that environment especially if families are around and kids and all that… I don't think the public would mind too much about that if you're just having a couple of drinks and you're not being too noisy, you're not being too out of control about it’. (P19) – ‘It depends on what they're doing. Like if they're jumping, what they're jumping from’. (P3)
Group membership and conformity	– ‘I think definitely people who are involved in like a like with their peers, like in a peer group of really young men definitely high risk of drinking, jumping. Being reckless around water. Oh yeah. I don't know yeah I feel like something you do, you don't do it by yourself so I feel like it's definitely more of a social situation’. (P14) – ‘It depends on like your state of mind like of relationships with them I suppose. Because if you're sober watching a bunch of drunk people at a pool party like we just look like a bunch of idiots. But if you're in there and you're in the atmosphere and you're joining in and everything like that it can actually be quite fun’. (P07) – ‘Well for the majority we'd consider that to be recklessness except people around that gender and age who are the targets of that attention seeking’. (P12) – ‘They (out‐group) might be a bit envious. Yeah, I know that when I see people doing things that I want to do, I wish I was doing that’. (P10)
Self‐identification	– ‘No. Because when we went swimming and stuff we were all quiet and we were just with ourselves. But when I pictured those types of people they're loud and they're boisterous, loud. People know that they're drunk’. (P11) – ‘Oh yes, yes so yes I was reckless when I did it and I did it to show off. And there was probably a girl. But I've done it but apparently it's not really my character and I've only done it under the influence of alcohol’. (P12) – ‘Like I would be one like I probably do, I'm a little bit reckless around water but I also like, I also can swim quite well and I've been around, I was a lifeguard for a while and kind of know, I know my limits and I think I know a bit more than like a lot of my friends do about water safety and stuff like that and how dangerous it can actually be. And how easy it is to drown… I'll do it to a point where I'll enjoy myself but I think I realise where it becomes dangerous’. (P14) – ‘I'd probably say I'm smarter than they are in terms of just not making stupid decisions. I could be biased but yeah I don't ever feel the need to try and do stupid things to make people laugh or anything like that. And also I'm not really a ‘rager’ as you might say like someone that gets drunk and just has a crazy time. Like I would prefer to just socialise and connect with people rather than go crazy’. (P21) – ‘Not really I'm… like I said I'm pretty confident with my water (?). Like I grew up pretty much in the water. I go to the beach every week we always had pools growing up everything like that. I'm very comfortable around water I'm just… like as a bartender I'm not that comfortable around drunk people plus water. Yeah so it's not so much for me. I know where my limits are and where I can and can't do certain things’. (P07)

### 3.1 Lived Experience of Consuming Alcohol Around Water

Within this broad category, several distinct themes were inductively derived from participants' descriptions of their experiences. These themes highlight the situational and contextual factors shaping how and when individuals consume alcohol around water.

#### 3.1.1 Social Context and Peer Encouragement

All participants indicated that consuming alcohol around water was typically a social activity performed in the company of friends and family: ‘Friends or family yeah. Pool party was friends, the boat was family, and then the beach was friends’ (P05, Age 18). Over half of the participants (*n* = 12) also recounted active encouragement from friends to drink and swim during these occasions: ‘When we went camping, we all just kind of decided to do it. The first time I was with basically an equal group of guys and girls and the guys kind of egged each other on and I think only two of the girls went in’ (P23, Age 21).

#### 3.1.2 Unplanned and Spontaneous Swimming

Many participants (*n* = 10) also described swimming after consuming alcohol as being unplanned, with one participant sharing ‘Yeah it was just a random kind of thing. I think I was wearing like jeans probably yeah’ (P08, Age 22), and another explaining ‘it's not always really planned but the opportunity is there. But yeah sort of just happens sometimes’ (P13, Age 20). Several participants (*n* = 7) indicated that drinking and swimming often occurs after partying, with one sharing ‘Usually it would just be if you're at a party and it happens to be close to the beach’ (P01, Age 23).

#### 3.1.3 Lack of Supervision and Safety Measures

Some participants (*n* = 5) reported consuming alcohol and swimming in areas without lifeguards or designated safety flags, often during times when these safety measures were not in place: ‘Usually I end up swimming at beaches that don't have lifeguards like or at times when there aren't lifeguards’ (P08, Age 22).

### 3.2 Risk Perception

All of the males reported an awareness of risk associated with swimming after consuming alcohol, recognising alcohol as a key contributor to the risk of drowning or injury around water. Despite acknowledging these general risks, participants' reflections on actual instances of drinking and swimming uncovered nuanced themes about their personal risk assessments and decision‐making processes. These themes revealed varied degrees of risk consideration, often showing a disconnect between participants' acknowledged awareness and their actions in those specific situations.

#### 3.2.1 Perceived Ability to Assess Risk and Evaluate Own Skills

Over half of the participants (*n* = 12) described confidence in their ability to handle potential risks and evaluate their own swimming skills when considering entering water after consuming alcohol. For example, participants described evaluating their level of intoxication, environmental conditions and personal swimming capabilities before deciding to enter the water. One participant shared, ‘I wasn't too concerned just because the environment didn't look too bad. It [the ocean] was calm and everything and I wasn't too concerned with my abilities that day’ (P19, Age 30). Some opted to stay in shallow areas or near the edge, assessing that engaging in more vigorous activities like swimming laps or heading into rough surf was too risky given their level of intoxication. Others based their decisions on familiarity with the location or the presence of safety measures like life jackets, with one participant sharing ‘we were all wearing life jackets. We were all you know, four drinks over three hours or four hours isn't really going to be too significant of an impact on how I think I'd be able to swim. Even prolonged swimming… trying to stay afloat for… ten, twenty minutes would…I think be fine’ (P05, Age 18). Swimming pools were also identified as low risk environments for swimming after consuming alcohol, in contrast to the unpredictable conditions of beaches: ‘I probably wouldn't have drunk alcohol and then went to the beach. But yeah in a commercial setting in a pool like that the risk of drowning was in my personal assessment very low’ (P05, Age 18).

#### 3.2.2 Consideration of Risk After the Event

Several participants (*n* = 7) described considering the risks of consuming alcohol and swimming only after the activity itself, with reflections on the potential dangers typically occurring retrospectively. For instance, one participant recounted a post‐event realisation of risk, stating, ‘Definitely after the fact. At the time it's kind of just like a fun like “Oh let's just do this”… And then after you sort of like “Oh that was probably… probably shouldn't have done that it's probably a bit risky”’ (P06, Age 22).

#### 3.2.3 Risk Not Considered

Many participants (*n* = 9) indicated that they did not actively think about the risks associated with consuming alcohol and swimming. Participants described engaging in water activities under the influence of alcohol without considering the potential dangers, as one participant explained ‘I don't know that I specifically thought about it [risk]…when I'm sober I can look at it objectively and be like, “I shouldn't do that”… But just at the time I guess I kind of wrote it off as being a symptom of being young and stupid’ (P23, Age 21). Another participant shared ‘I don't think I've consciously thought “Oh I am a bit intoxicated I shouldn't swim, I should be more cautious”’ (P14, Age 22).

### 3.3 Social Identity

Several themes were derived from participants' responses regarding their perceptions of the types of people who consume alcohol around water, including whether these behaviours were characteristic of specific types of people or age groups, the social desirability of such behaviours, how closely participants identified with these groups, and their self‐perception when engaging in alcohol consumption around water.

#### 3.3.1 Characteristics of Typical Individuals Who Consume Alcohol Around Water

Participants described what they believed to be the characteristics of a typical individual who consumes alcohol around water. They described these individuals as primarily male (*n* = 14), and predominantly ranging in age from teenagers to about 30‐year‐old (*n* = 19). Participants also identified these individuals as being sensation‐seeking in nature (*n* = 13), using descriptors like ‘adventurous’, ‘risk‐taker’, ‘attention‐seeker’, ‘reckless’ and ‘extraverted’. One participant described ‘Yeah more adventurous definitely. Especially when they're like not swimming exactly. Like boating or jet skiing or wakeboarding or anything like that? Yeah definitely more adventurous’ (P02, Age 30), and another described ‘I guess maybe [they] might be show‐offs or they might take risks. They might take risks normally. So if they're around the pool they might yeah take extra risks or something’. (P03, Age 27).

#### 3.3.2 Perceptions of Social Desirability

The majority of participants (*n* = 17) identified consuming alcohol around water as a socially undesirable behaviour, describing people who engage in alcohol consumption around water as being perceived as irresponsible, foolish and reckless, attracting social disapproval particularly when they pose a risk to others, for example when boating or using watercraft. One participant stated, ‘It's just irresponsible stupid you know careless. It's selfish as well I guess if you've consumed alcohol and you're taking control over something or have, you know power this or whatever. Then that's you know pretty selfish considering you do have people's lives in your hands’. (P04, Age 28). Some participants (*n* = 7) perceived the social desirability of consuming alcohol around water as neutral or even positive, describing it being perceived as adventurous, a culturally iconic activity, or a normal part of relaxed social gatherings. As one participant shared, ‘it's part of our culture, like it feels like an iconic Australian thing to do is have a barbie have some beers have a swim or like have a surf…it's probably like the ideal environment for hanging out with your friends, I would say’ (P21, Age 22).

Moreover, a few participants (*n* = 4) specified that social disapproval is focused more on overt public rowdiness and visible drinking/drunkenness in public, suggesting that quieter, less conspicuous consumption of alcohol around water does not attract negative attention unless it becomes disruptive or clearly irresponsible in public settings. As one participant explained ‘I think if it's the type that are drinking heaps and getting really rowdy with a big bunch of people I think people would not perceive them really well… I don't think the public would mind too much about that if you're just having a couple of drinks and you're not being too noisy, you're not being too out of control about it’ (P19, Age 30).

#### 3.3.3 Group Membership and Conformity

Although the majority (*n* = 17) perceived disapproval from the general public regarding the consumption of alcohol around water, several participants (*n* = 6) noted that within their immediate peer groups or in‐groups, such behaviours often receive encouragement, especially among like‐minded individuals who share similar risk‐taking tendencies, whereas out‐groups—people who do not belong to the group an individual identifies with—were more inclined to perceive these actions as reckless or irresponsible. One participant described how group dynamics shape these perceptions: ‘It depends on like your state of mind like of relationships with them [individuals drinking and swimming] I suppose. Because if you're sober watching a bunch of drunk people at a pool party like we just look like a bunch of idiots. But if you're in there and you're in the atmosphere and you're joining in and everything like that it can actually be quite fun’ (P07, Age 19). Another participant further illustrated how conformity within specific social demographics, such as age and gender, shapes what is viewed as acceptable: ‘Well, for the majority we'd consider that to be recklessness, except people around that gender and age who are the targets of that attention seeking’ (P12, Age 21).

#### 3.3.4 Self‐Identification

Although all participants had previously consumed alcohol in, on and around water, many did not see themselves as embodying the typical characteristics of such individuals (*n* = 8). Participants differentiated themselves by describing their behaviour as more subdued or responsible compared with the stereotypical loud, boisterous and reckless drinker‐swimmer portrayed in their descriptions. As one participant explained ‘… when we went swimming and stuff we were all quiet and we were just with ourselves. But when I pictured those types of people they're loud and they're boisterous, loud. People know that they're drunk’. (P11, Age 23), and another participant shared ‘I'd probably say I'm smarter than they are in terms of just not making stupid decisions. I could be biased but yeah I don't ever feel the need to try and do stupid things to make people laugh or anything like that’ (P21, Age 22).

## 4 Discussion

Drowning is a significant public health threat, particularly affecting young males, and especially when alcohol is consumed in aquatic settings [1, 5, 32]. Building on prior research into alcohol use and water‐based activities [33, 34], this qualitative study provides unique insights by focusing on the firsthand lived experiences, risk perceptions and social identity dynamics of young males who have actively engaged in the behaviour of drinking and swimming. By drawing directly from the real‐world experiences of this target population, the study offers a deeply contextualised understanding of how individuals perceive and navigate the risks and social contexts of this behaviour. Thematic analysis revealed key insights into participants' lived experiences, their perceptions of risk and the role of social identity, providing a foundation for future research to inform more targeted strategies for harm reduction.

The insights garnered from participants' lived experiences highlight the often social and spontaneous nature of young males consuming alcohol around water. Participants consistently reported that activities involving alcohol consumption around water typically occur in the company of friends and family, are often spontaneous and unplanned, and frequently take place in locations lacking formal safety measures such as rivers or at unpatrolled beaches or beaches after such time as patrols have ceased. In response, fostering a collective sense of responsibility for each other's safety among all group members may be particularly effective. Such an approach may appeal to subjective masculine norms such as ‘leadership’ and ‘protection of others’ [35] thereby enhancing its acceptance and engagement within this demographic. Another effective strategy could be to implement educational programs in schools and universities that leverage the real‐world lived experiences of young males to challenge myths and address misconceptions about the safety of their behaviours. This could involve incorporating case studies and testimonials from peers who have experienced real consequences of consuming alcohol around water, highlighting the discrepancies between perceived safety and actual risk and challenging optimism bias. Such an approach could be especially impactful in this demographic, given that many males in this study perceived their own consumption of alcohol around water as responsible, whereas deeming others' actions as less so. Additionally, a targeted educational campaign designed to shift social norms and portray the combination of alcohol consumption and aquatic activity as socially unacceptable could effectively promote safer behaviours. For example, some literature has recommended education through mediums such as social media and messaging, which employs humour or shock tactics [36], though no strategies such as these have yet been evaluated in the current context.

Second, addressing the spontaneous and unplanned nature of drinking and swimming can be managed through proactive planning strategies, such as implementation intentions [37, 38]. For instance, if hosting a social gathering around water, planning water activities early in the day and transitioning to non‐water activities before alcohol is served could help to mitigate unplanned drinking and swimming. Additionally, planning safe routes home from a night out that avoid tempting locations, such as beachfronts, could prevent impulsive decisions to swim while intoxicated. This type of forward planning has demonstrated effectiveness in changing driver intentions to engage in risky driving behaviour such as driving into urban floodwaters [39]. This approach may be particularly effective for males, redirecting impulsivity—often observed in male risk‐taking [40]—into more deliberate, measured decision‐making. At last, increasing the presence of safety information at points where alcohol is sold near water bodies and enhancing signage and physical safety features at popular swimming locations could mitigate risks. Establishing alcohol‐free zones at various aquatic locations [41], beyond just beaches and ensuring strict enforcement of these zones, as well as restricting alcohol outlets near water [42], can further support safety efforts [41]. These measures, coupled with educational campaigns, can encourage safer practices in relation to consuming alcohol around water.

The findings for risk perception revealed young males are aware of the dangers associated with consuming alcohol around water, yet this awareness does not always translate into safe behaviours. Nearly half of the participants believed they could accurately assess their risk and abilities when deciding to swim after drinking, which suggests a gap between their perception of safety and the actual risks involved. This aligns with previous research showing that men often overestimate their abilities and underestimate actual risks in aquatic activities [43]. Compounding this issue, research indicates that perceived susceptibility and severity are poor predictors of young males' intentions to drink and swim [10], suggesting traditional risk communication may be inadequate. Additionally, the effectiveness of risk interventions heavily depends on individuals recognising their vulnerability to these risks [44], a perception that appears low in this population. Notably, all the young males we interviewed self‐rated their swimming ability as at least average, with most classifying it as good or better—findings consistent with previous research reporting self‐rated swimming confidence in young adults [11]. Such potentially inflated confidence in their swimming proficiency may limit the efficacy of risk communication and intervention strategies, as it could reduce the salience of safety messages and diminish young males' motivation to adopt safer behaviours.

To strengthen males' perceptions of risk towards consuming alcohol around water, future campaigns could employ more persuasive communication techniques in their messaging [45], especially to address young males' optimism bias regarding personal risk [46] and their potentially inflated confidence in their swimming ability. Furthermore, interventions targeting young males should clearly communicate how alcohol consumption impairs not only risk judgement but also actual swimming ability [47, 48], not just the ability to assess risk. We propose that public health campaigns reinforce this by directly connecting drinking with decreased swimming capability. The strategy is supported by research showing that awareness of lower swimming proficiency is linked with reduced intentions to engage in drinking and swimming among young males [10]. Effective approaches could include scenario‐based risk information delivered through interactive simulations to illustrate alcohol's physical impairments [49]. Such methods could help young males reassess their capabilities while intoxicated and encourage safer behaviour by reshaping their perceptions of risk associated with alcohol and water activities.

Additionally, with some participants only considering the risks of consuming alcohol around water post‐activity, and others not at all, there is a clear need for interventions targeting decision‐making processes prior to the activity occurring. For example, distributing campaign materials at popular water locations before peak times for alcohol‐related drownings, such as public holidays and summer [6, 50]. Furthermore, strategically placing campaign content on websites and apps frequently visited by young males before engaging in drinking and swimming activities, like weather forecasts.

Regarding social identity, there was agreement on the typical characteristics of individuals who consume alcohol around water: young, male and often described as ‘adventurous’, ‘risk‐taker’ and ‘reckless’. This characterisation is supported by research linking ‘sensation‐seeking’ with swimming after consuming alcohol [42]. Although there was consensus on these characteristics, perceptions of social desirability varied. The majority viewed consuming alcohol around water as socially undesirable, labelling it irresponsible and foolish. However, in line with prior studies, a subset of participants saw drinking around beaches, pools and waterways as a culturally iconic activity [33, 42]. To strengthen the perception of social undesirability and reduce the tendency to rationalise or distance themselves from ‘drinker‐swimmers’, useful strategies could include providing information about others' disapproval and creating opportunities for social comparison [49].

Furthermore, discussions about group membership and conformity revealed that within these young males' peer groups, such behaviours might not only be accepted but admired. This underscores the important role of cultural norms, group norms and social identity as underpinning decisions related to alcohol consumption and water safety, aligning with existing research [34]. Previous public health interventions, such as Royal Life Saving–Australia's safety campaign ‘Don't let your mates drink and drown’, have targeted these dynamics, demonstrating short‐term effectiveness at influencing males' intentions, subjective norms and attitudes towards discouraging mates from drinking and swimming, and drinking and swimming themselves [51]. This campaign targeted men aged 25–34 years, using the fictional character ‘Dave’—a model of responsible behaviour—to encourage participants to protect their friends around water and intervene against risky behaviours and to see themselves as part of a responsible group that values safety. However, the effects of such campaigns have not proven to be long‐lasting [51], indicating a need for strategies that promote sustained change. Interventions informed by the beliefs, lived experiences and social contexts of the target group, alongside co‐design with the target population, could enhance their relevance and effectiveness, thereby fostering long‐term improvements in water safety behavioural intentions.

Finally, despite agreeing on the stereotypical traits of individuals who engage in risky water behaviours—such as being reckless and adventurous—many participants described their own actions as more responsible, highlighting a dissonance between their self‐perception and actions. To leverage this in public health campaigns targeting drinking and swimming among young males, strategies should highlight the conflict between individuals' responsible self‐image and their risky behaviours [45]. Using testimonials or real‐life scenarios, campaigns should challenge viewers to assess whether their actions match their self‐identity as safe and sensible, thus encouraging alignment of behaviours with positive self‐perceptions to improve water safety. Furthermore, given the importance of peer norms among young males, real‐life stories of individuals who misjudged their risk could serve as powerful examples that prompt self‐reflection and encourage safer practices. For example, a campaign could feature stories from individuals who believed they were drinking and swimming responsibly but encountered dangerous situations, prompting viewers to reflect on their own behaviours and make safer choices.

### 4.1 Study Limitations

Although this research offers important insights for intervention design and future studies, it is important to acknowledge several limitations. First, the qualitative design may introduce participant bias, as it depends on individuals' ability to recall and reflect on their experiences, which may not always be accurate [52]. Additionally, the thematic analysis employed could be subject to biases, such as researchers' preconceptions or prior knowledge affecting theme selection and interpretation. However, to counteract this, member checking was integrated into the interview process, allowing for immediate validation by participants, which strengthened the reliability of the resulting thematic conclusions. Examining only individuals who have engaged in alcohol consumption around water may introduce bias in how participants perceive and describe factors relevant to social identities associated with this behaviour, potentially limiting the diversity of perspectives. Participant responses may also have been shaped by social desirability bias due to the sensitive nature of the topic [53], particularly given the affiliation with Royal Life Saving Society–Australia, which was disclosed during recruitment and on the information sheet sent to participants. The choice of a female interviewer was deliberate to prevent participants from presuming shared understanding with an interviewer of the same demographic, which could limit their explanations. However, without data comparison from a male interviewer, the full impact of interviewer gender remains uncertain. Moreover, the interviews took place during COVID‐19 restrictions in Australia, possibly affecting the recency of participants' experiences and their responses. The pandemic also influenced the data collection efficiency and mode, although telephone interviews have been found to enhance openness and reduce perceptions of judgement [48]. With respect to self‐reported swimming ability, all participants rated their proficiency as average or better. Although it is possible that high perceived swimming ability is characteristic of the demographic under study, it may not represent individuals with a broader range of self‐perceived abilities. Finally, although self‐reported swimming ability was collected, the study was not designed for subgroup analysis. Namely, we did not purposively recruit comparable subgroups based on swimming ability. Future research incorporating a broader sample and analytical framework to compare different perceived ability groups could further inform tailored intervention strategies and improve the generalisability of findings.

### 4.2 Conclusions

The current study employed a qualitative approach to explore the lived experiences, risk perceptions and social identities of young males related to consuming alcohol around water. Through the analysis of interviews with 23 males who had previously engaged in this behaviour, the study uncovered rich insights into how these factors shape their decisions. Findings support the development of strategies that specifically leverage the lived experiences, perceived risks and social identity influences that affect young males' decisions to consume alcohol in aquatic settings. Participants' accounts suggest that young males consuming alcohol around water often do so in social, spontaneous settings, frequently lacking formal safety measures. Although they acknowledge potential dangers, a gap appears between their awareness and actions, possibly influenced by inflated self‐perceived swimming ability and peer dynamics. These findings indicate that interventions may benefit from promoting strategies like designating a ‘responsible mate’, incorporating scenario‐based education, and clearly conveying how alcohol impairs both judgement and swimming proficiency. Additionally, emphasising the discrepancy between individuals' self‐image and their risky behaviours and challenging optimism bias, may encourage more cautious decision‐making. Such approaches have the potential to enhance the relevance and efficacy of public health campaigns aimed at improving water safety behaviours among young males. These insights, garnered from individuals with direct experience and representing the most at‐risk group, provides a foundation for further research aimed at developing more effective strategies to reduce alcohol‐related harm and improve safety in aquatic environments.

## Ethics Statement

This project received ethical approval from the Griffith University Human Research Ethics Committee (ref no: 2019/294).

## Conflicts of Interest

The authors declare no conflicts of interest.

## Data Availability

The data that support the findings of this study are available on request from the corresponding author. The data are not publicly available due to privacy or ethical restrictions.
